# Haemophagocytic Lymphohistiocytosis with Leptospirosis: A Rare but Devastating Complication

**DOI:** 10.1155/2021/3451155

**Published:** 2021-07-08

**Authors:** B. M. Munasinghe, A. G. Arambepola, N. Pathirage, U. P. M. Fernando, N. Subramaniam, S. Nimalan, T. Gajanthan

**Affiliations:** ^1^Department of Anaesthesia and Intensive Care, District General Hospital, Mannar, Sri Lanka; ^2^National Blood Transfusion Service, Colombo, Sri Lanka; ^3^Department of Medicine, District General Hospital, Mannar, Sri Lanka

## Abstract

**Introduction:**

Secondary haemophagocytic lymphohistiocytosis (sHLH), often associated with an array of infections, malignancies, and autoimmune diseases, is rarely seen with leptospirosis, which carries a relatively poor prognosis even with modern state-of-the-art medical care. We describe a patient with leptospirosis complicated by sHLH who succumbed to illness following multiorgan dysfunction. *Case Description*. A 74-year-old farmer presented with high-grade, unsettling fever for a week. Muddy water exposure and suggestive symptoms prompted investigation and management in the line of leptospirosis (IV ceftriaxone was instituted, and later, MAT (microscopic agglutination test) became positive). Subsequently, he developed severe acute hypoxemia requiring mechanical ventilation and acute renal failure requiring renal replacement therapy. Bone marrow biopsy and markedly elevated serum ferritin and triglyceride levels done on day 10 (with unresolving fever, hepatosplenomegaly, and pancytopaenia) confirmed the diagnosis of HLH. The routine cultures, retroviral studies, CMV, dengue, hanta and mycoplasma antibodies, tuberculosis and COVID-19 PCR, and malaria screening were all normal. There was no improvement of hypoxemia following intravenous methylprednisolone. He died on day 15 despite escalating organ support.

**Conclusion:**

Leptospirosis is a common zoonotic disease in the tropics with significant morbidity and mortality. In the case of severe leptospirosis, overlapping clinical features with sHLH make the diagnosis of the latter challenging. No assessment tools are available to date to predict the risk of developing sHLH in a patient having leptospirosis. Outcome following sHLH due to leptospirosis still remains majorly ominous. A high index of suspicion and low threshold for specific investigations could possibly alter the outcome following such an occurrence.

## 1. Introduction

First described in 1939 [1], haemophagocytic lymphohistiocytosis (HLH) still evades the diagnosis of contemporary physicians [2] and carries a high mortality. Categorized as primary and secondary, the latter could affect any age group and is linked with an array of infections. Secondary HLH (sHLH) due to leptospirosis is rarely documented in the literature. Here, we present a case of a HLH secondary to leptospirosis in an elderly South Asian male, who sought medical help a week into his illness and had a poor outcome. A detailed literature review on similar reported cases is also included in the text.

## 2. Case Report

**Figure 1:**
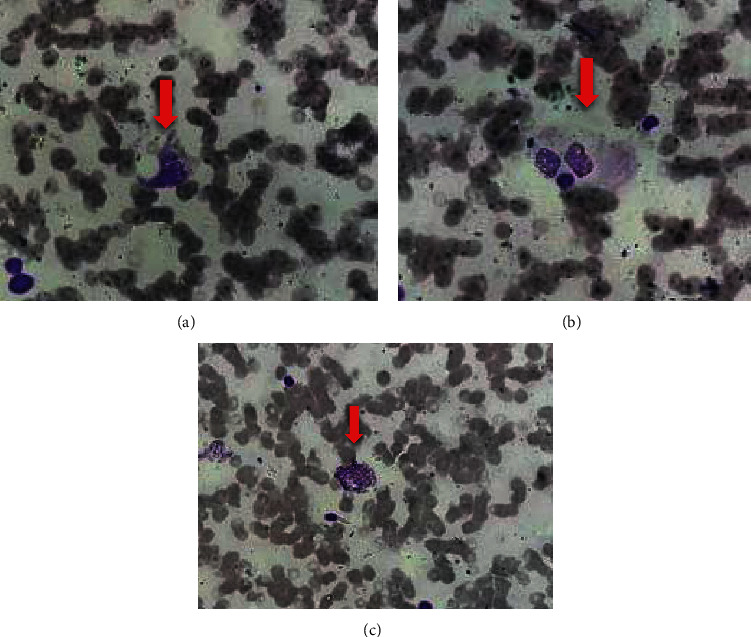
(a–c) Bone marrow (trephine) biopsy illustrating haemophagocytes (red cell-engulfed macrophages), shown in red arrows. H&E stain (10 × 40).

A 74-year-old known diabetic and hypertensive with good control presented to our institution with unsettling, high-grade fever for a week associated with retro-orbital headache, arthralgia, and myalgia. He was a farmer by profession and has had contact history with muddy water. He did not have respiratory or urinary symptoms. He was haemodynamically stable initially although urine output was low (<0.5 ml/kg/hr). Renal functions were markedly elevated, and bilateral moderate pleural effusions were noted in the ultrasound chest. Glycemic control was poor with initial random blood sugar of 350 mg/dl requiring an insulin infusion. Chest X-ray showed additional features of pulmonary haemorrhages. There were no skin rashes or lymphadenopathy. Blood was collected for routine cultures and *Leptospira* antibody test (microscopic agglutination test, MAT). He was started on intravenous ceftriaxone and methylprednisolone empirically with the suspicion of severe leptospirosis and pulmonary haemorrhages. Urgent haemodialysis was arranged every other day. Two days into the hospital stay, the patient acutely became drowsy and restless and electively intubated. Noncontrast CT brain excluded cerebral oedema or acute vascular events. Arterial blood gas analysis revealed a severe hypoxemia (P/F < 120). Lung-protective ventilation was continued although a fraction of inspired oxygen continued to escalate. Prone ventilation was opted approximately 14 hours a day. Pancytopaenia with hepatosplenomegaly was evident in the complete blood count and ultrasound abdomen, respectively. A multidisciplinary discussion with the general medical and haematological units was held. Persistent high fever with negative routine cultures and the rapid deterioration despite optimal supportive therapy pointed towards possible sHLH. Subsequent serum ferritin levels and trephine biopsy confirmed the diagnosis (Figures 1(a)–1(c)), aided by elevated serum triglycerides. After three doses of intravenous methylprednisolone, intravenous dexamethasone was commenced with intravenous etoposide twice a week. Sequential blood pictures and clotting parameters were suggestive of disseminated intravascular coagulation (DIC). This was managed with transfusion of cryoprecipitate and fresh frozen plasma per haematological guidance. Persistent severe metabolic acidosis and severe hyperkalaemia were treated with haemodialysis under escalating inotropic supports (continuous renal replacement therapy was not available in our center). No structural anomalies or vegetations were detected in the 2D echocardiogram. The patient died following cardiac arrest on day 06 of the intensive care (day 15 of the illness). A highest MAT titre of 1 : 640 confirmed the diagnosis of leptospirosis posthumously. The summary of investigation results is illustrated in Table 1.

## 3. Discussion

Haemophagocytic lymphohistiocytosis (HLH) is a rare but possibly fatal, rather underdiagnosed disease state, affecting both adults and children [3]. Amidst the rarity, a preponderance towards middle-aged (49 years) males (1.7 : 1) has been described recently among adults [2]. Primary and secondary variants are widely described in the literature. The secondary HLH, otherwise referred to as sHLH, acquired or reactive HLH, has been mainly associated with infections, malignancies, rheumatologic disorders, pregnancies, and drugs [4]. Viral infections (mainly CMV and EBV) predominate although bacterial, fungal, and parasitic pathogenesis have been described in the literature under infectious triggers. Bacteria with a nonexhaustive list are frequently associated with sHLH in East and South Asian countries [5]. By 2020, around 70 reported cases of sHLH due to *Mycobacterium tuberculosis* are found in the literature, the leading bacterial infection of sHLH [4]. Interestingly, being a common zoonotic disease in the tropics, the reported cases of *Leptospira*-induced sHLH are remarkably few. The reported cases are tabulated in comparison to our patient in Table 2.

The proposed mechanisms and models of HLH led organ dysfunction describe dysregulated T cell activation (mainly CD8+ cytotoxic T cells) which is propagated with macrophages, leading to a ‘cytokine storm.' It is postulated that the pathophysiology of infection-associated HLH includes excessive stimulation of Th1-mediated immune response resulting in the overproduction of tumour necrosis factor alpha (TNF-*α*), interleukin 1 or interleukin 6, and gamma interferon [11]. This may be true for most intracellular pathogens, but *Leptospira,* being commonly extracellular, a genetic predisposition of the organism to utilize and internalize host haem proteins, could possibly contribute to its pathophysiology and HLH [13, 14]. It is hypothesized that *Leptospira* might induce a TNF-*α*-associated apoptosis of macrophages, excessive secretion of interleukins, and ultimately cytokine storm and multiorgan failure [15], even though the exact mechanism of sHLH is still unclear.

The diagnostic criteria for HLH were mainly intended for the paediatric population and primary HLH. According to subsequent updates (HLH-2004), at least five out of eight criteria should be met to establish the diagnosis [16]. While HLH-2004 is continued to be utilized in diagnosing sHLH in adults in most parts of the world, it has few drawbacks. The cutoff values of the included criteria are varied [17]. For developing countries, sophisticated cytokine or cell activity assays are virtually nonexistent, or reports are delayed elsewhere. Serum ferritin levels, though sensitive, are not specific as elevated levels are seen in other conditions [18]. To address these issues, H-scoring was proposed as a probability score of sHLH [17]. The H-score adopts variables which are weighted [17]. A cumulative value of 169 is demonstrated to have a sensitivity and specificity of 93% and 86%, respectively.

The management of sHLH is mainly in the aspects of controlling the trigger, chemotherapy, and immunotherapy with supportive care, frequently comprising the specialties of haematologists, oncologists, rheumatologists, microbiologists, and intensivists. In the case of leptospirosis-induced sHLH, early intravenous antibacterial therapy (with penicillin group or third-generation cephalosporins) should be instituted as patients are severely ill. According to the literature review (Table 2), out of the eight recorded cases, four had developed acute kidney injury, three requiring renal replacement therapy. Other organ supports may include mechanical ventilation or supplementary oxygen therapy (depending on the pulmonary involvement, conscious level, and severity of the cardiorespiratory compromise), correction of coagulopathy using blood products [3], and vasopressor or inotropic therapy for persistent hypotension. Even though the evidence for the use of high-dose corticosteroids in the case of severe pulmonary manifestations is heterogeneous and at best is inconclusive [19], they are still being used, rationalized by the hyperactive immune state in both severe leptospirosis and sHLH following leptospirosis. Six out of the eight patients described here were given corticosteroids as either methylprednisolone, dexamethasone, or prednisolone. Out of the patients who survived (four), three had received corticosteroids, and two were children. Thus, there may be a beneficial effect of corticosteroids in this cohort of patients. Intravenous immunoglobulin is suggested as a joint therapy with corticosteroids once the infection is being abated [20]. When the outcome is considered, the survivors of this review had lengthy hospital stays varying from 21 days to 35 days, requiring organ supports. This implies increased costs associated with this potentially fatal condition. Apart from one survivor whose biochemical parameters have been normalized at one year, follow-up data of the rest of the survivors were unavailable.

The diagnosis of sHLH in intensive care units poses a significant challenge to physicians due to unspecific symptoms which may overlap between conditions such as sepsis, multiorgan dysfunction syndrome, or other cytokine storm syndromes and coexistence of these conditions [8, 21]. Moreover, sepsis can also act as a trigger for sHLH. The fatal outcomes in intensive care patients with HLH are higher where late diagnosis and late commencement of HLH-specific therapy and organ failure play a crucial role.

In the case of our patient, who presented late with acute kidney injury and probable setting severe adult respiratory distress syndrome, sHLH was suspected immediately after admission to the intensive care unit with pancytopaenia and rapid deterioration and pulsed corticosteroid therapy as intravenous methylprednisolone was commenced with other organ supports. The steroid therapy was continued with dexamethasone. The patient had already been started on third-generation cephalosporin on admission targeted for *Leptospira*, the response to which was assessed by means of serial CRP as procalcitonin was not available in our center. Even though there was a rise in CRP, which could have been due to the generalized inflammatory process, we opted for intravenous etoposide following hematology consultation due to poor response to steroid therapy. In comparison to the HLH-94 treatment protocol which is utilized primarily for paediatric HLH, modified, individualized HLH-94-like treatment protocols have been suggested for adult sHLH, although caution is warranted for immunosuppression following intracellular organism-related infections as resolution of HLH has been associated with specific antimicrobials alone [21].

By this review, it is evident that the outcome of leptospirosis-induced sHLH is poorer in adults in comparison to the paediatric age group. Associated comorbidities might play a role in accentuating or altering the immune response and accelerating the organ dysfunction; however, late diagnosis and nonadherence to the current guidelines during management are crucial factors contributing to these figures. It is likely that sHLH in association with leptospirosis may be misdiagnosed as severe leptospirosis due to the overlapping constellation of symptoms and signs, persistent fever, organomegaly, and cytopenia, making the diagnosis of sHLH underreported.

Leptospirosis is considered as a neglected but reemerging disease throughout the world [22]. A systematic review by Warnasekara et al., in 2018, estimated an annual incidence of 300·6 (95% CI 96·54–604·23) per 100,000 people in Sri Lanka with a case fatality ratio of 5.3% in selected intensive care units [23]. The same study points out that the deaths are mostly due to multiorgan failure, mainly involving the renal and pulmonary systems. Out of the reported literature, this was interestingly the first case of sHLH associated with leptospirosis in the country. With regard to the associated complications throughout the world, they are varied and invariably associated with increased mortality and morbidity. This is especially true for sHLH, where the mortality among adults was found to be 80% in this brief review. For a patient with leptospirosis who does not respond to optimal antibiotics and with rapid deterioration and multiorgan dysfunction, a high index of suspicion is warranted for sHLH as early involvement of panel of subspecialties, intensified investigations and monitoring, targeted therapy with corticosteroids and/or immunoglobulin could possibly alter the outcome.

## 4. Conclusion

Leptospirosis will continue to burden the developing nations with increased morbidity and mortality. Even though being a rare association, most probably due to underdiagnosis and/or misdiagnosis, sHLH, due to severe leptospirosis, may also contribute to these figures. No assessment tools are available to date to predict the risk of developing sHLH in a patient having leptospirosis. When dealing with leptospirosis-induced sHLH, a high index of suspicion, especially in cases of abrupt deterioration of the clinical condition and poor response to standard therapy, is decisive to escalate the treatment protocol. Nonetheless, early diagnosis of sHLH by devising proposed scoring systems, early ICU care, and timely institution of corticosteroid therapy and immunosuppressant therapy, whenever appropriate, may dramatically improve the patient outcome as evident by this work.

## Figures and Tables

**Table 1:** Sequential investigation results during intensive care stay.

Investigation	D 01	D 02	D 03	D 04	D 05	D 06	Reference range
Hb	9.5	8.3	8.7	7.8	7.8	8.0	10–14 g/dl
PCV	27.5	25.2	25.7	24.7	23.8	24.1	34–45
Platelet	15	5	15	41	38	20	150–450 × 10^3^/mm^3^
WBC	4.9	14.8	11.6	7.6	5.8	0.9	4–11 × 10^3^/mm^3^
Neutrophils	3.5	13.2	10.6	6.9	5.3	0.74	2–8 × 10^3^/mm^3^
Lymphocytes	1.1	0.8	0.26	0.29	0.2	0.16	1–5 × 10^3^/mm^3^
ESR	60	—	50	—	—	—	0–20 mm/h
PT	25.5	22.2	19.6	19.8	16.8	21	11–12.5 s
INR	2.23	2.33	1.62	1.63	1.3	1.73	08–1.1
APTT	—	45.1	41.7	24.9	42.2	32.8	30–40 s
Na^+^	145	144	138	136	185	132	135–145 mmol/l
K^+^	4.3	4.6	7.06	6.58	6.02	6.04	3.5–4.5 mmol/l
Ca^2+^	1.2	1.3	1.1	1.4	0.8	1.2	1.1–1.3 mmol/l
B. urea	311	268	294	329	381	384	18–55 mg/dl
S. creatinine	10.8	10.7	11.2	11.2	12.8	12.4	0.7–1.3 mg/dl
ALT	615	780	550	262	127	124	0–45 IU/L
AST	1780	2754	1039	515	365	352	0-35 IU/L
ALP	178	—	—	—	—	—	80–300 U/L
Gamma GT	180	—	—	—	—	—	9–48 U/L
Total bilirubin	6.6	6.4	6.8	5.4	5.5	6.7	0–1.4 mg/dl
Direct bilirubin	0.3	6.0	6.2	4.8	4.9	6.2	0–0.3 mg/dl
Indirect bilirubin	6.3	0.4	0.6	0.6	0.6	0.5	—
Serum amylase	234	—	—	—	—	—	30–110 U/L
CRP	141	—	165	—	228	—	<6 mg/L
S. ferritin	—	60685	—	—	—	—	20–250 ng/ml
LDH	—	450	—	—	—	—	150–300 u/L
Total cholesterol	—	168	—	—	—	—	<200 mg/dl
Triglycerides	—	358	—	—	—	—	<150 mg/dl
LDL	—	82	—	—	—	—	<100 mg/dl

Troponin T	Negative	—	—	—	—	—	—
UFR	—	—	—	—	—	—	—
Albumin	2+	—	—	—	—	—	—
Pus cells	12–18/HPF	—	—	—	—	—	—
Red cells	Nil	—	—	—	—	—	—
Urine ketone bodies	Negative	—	—	—	—	—	—
Urine culture	—	No growth	—	—	—	—	—
Blood culture	—	No growth	—	—	—	—	—

Serological studies							
Malaria screening	—	Negative	—	—	—	—	—
Hanta antibodies	—	Negative	—	—	—	—	—
Mycoplasma antibodies	—	Negative	—	—	—	—	—
CMV antibodies	—	Negative	—	—	—	—	—
EBV antibodies	—	Negative	—	—	—	—	—
TB PCR	—	Negative	—	—	—	—	—
COVID-19 PCR	—	Negative	—	—	—	—	—
*Leptospira* antibody test (MAT with the pathogenic *Leptospira* panel)	—	1 : 640 (significant result)	—	—	—	—	—

Blood picture	Initial: marked thrombocytopenia and rouleaux formation, no features of haemolysis, exclude DIC						
	Later: suggestive features of DIC						

Bone marrow trephine biopsy	Haemophagocytes present, suggestive of HLH, no additional abnormalities in the main cell lineages						

USS abdomen	Mild hepatosplenomegaly, bilaterally increased renal echogenicity suggestive of acute kidney injury, no intra-abdominal collections or other organomegaly						

Hb: haemoglobin; PCV: packed cell volume; WBC: white blood cells; ESR: erythrocyte sedimentation rate; PT: prothrombin time; INR: international normalized ratio; APTT: activated partial thromboplastin time; ALT: alanine aminotransferase; AST: aspartate aminotransferase; ALP: alkaline phosphatase; CRP: C-reactive protein; UFR: urine full report; HPF: high-power field; CMV: cytomegalovirus; EBV: Epstein–Barr virus; TB PCR: tuberculosis polymerase chain reaction; MAT: microscopic agglutination test; LDH: lactate dehydrogenase.

**Table 2:** Review of reported cases of sHLH following leptospirosis.

	Yang et al. [6]	Sripanidkulchai and Lumbiganon [7]	Rajagopala et al. [8]	Krishnamurthy et al. [9]	Kodan et al. [10]	Barman et al. [11]	Jevtic et al. [12]	Our patient (2021)
Age (years)	61	8.6	53	4	24	40	13	74

Sex	Female	Male	Female	Male	Male	Male	Female	Male

Country	Taiwan	Thailand	India	India	India	India	Serbia	Sri Lanka

Comorbidities	Type 2 DM for 4 years, psoriasis vulgaris for 10 years	NA	NA	None	NA	None	None	Type 2 DM, hypertension

Presentation	4-day history of abdominal pain, malaise, diarrhoea, and intermittent fever	Fever >37.8°C for more than 6 days	Fever, delirium, purpura for 10 days	High-grade fever for 7 days	Fever and oliguria	High-grade fever for 10 days, yellowish discolouration of the eyes and urine for 4 days, recurrent nasal bleeding for 2 days	A 20-day history of fever, swelling, and neck pain following a rash	High-grade fever for 7 days

Diagnostic criteria for sHLH	Fever: 39°C, splenomegaly, WBC: 1900, Hb: 10.1, TG-: 414, S. ferritin: 9152, BM biopsy: HS	Fever, leukopenia, thrombocytopenia	Fever, organomegaly, TG: 366, S. ferritin: 16,192, BM aspiration: HS	Fever, organomegaly, TG: 658, S. ferritin: 1329, BM biopsy: HS	Fever, organomegaly, cytopaenia, S. ferritin: 6360, BM biopsy: HS, TG: NA	Fever >39°C, organomegaly, WBC: 1500, Hb: 6.5, TGL 525, S. ferritin: 1400, BM biopsy: HS	Fever, organomegaly, Hb: 9.4, Plt: 63,000, S. ferritin: 1500, BM biopsy: HS, soluble interleukin-2R alpha	Fever >38.5 C, Hb: 7.8 g/dl, Plt <40,000, organomegaly, S. ferritin: 60,685, TG: 358, BM biopsy: HS

Management	Ceftriaxone, metronidazole, day 07: oral doxycycline, intermittent haemodialysis	NA	Steroids, antimicrobials, mechanical ventilation, vasopressors	Ceftriaxone and prednisolone	Corticosteroids	Ceftriaxone, doxycycline, methylprednisolone	Meropenem and cefuroxime, IVIG, corticosteroids, haemodialysis	Ceftriaxone, methylprednisolone/dexamethasone, etoposide, intermittent haemodialysis, lung-protective ventilation

Complications	AKI deteriorating liver functions, generalized seizures, coma	Ascites, pericardial effusion, cardiogenic shock	AKI, ARDS, fulminant hepatic failure	NA	NA	Refractory hypotension, pancytopaenia, progressive hypoxemia requiring mechanical ventilation	AKI	AKI, severe ARDS, pulmonary haemorrhages

Number of hospital days	12	21	7	3 weeks	NA	12	35	08

Outcome	Death	Responded to therapy	Death following CRBSI	Responded well to the treatment; haematological and biochemical derangements normalized at one year	Responded to therapy	Death following cardiac arrest	Full recovery	Death following cardiac arrest

DM: diabetes mellitus; NA: not available; WBC: white blood cells; Hb: haemoglobin; TG: triglycerides; Plt: platelets; S. ferritin: serum ferritin; BM biopsy: bone marrow biopsy; HS: haemophagocytes; CRBSI: catheter-related blood stream infection; IVIG: intravenous immunoglobulin; AKI: acute kidney injury; ARDS: adult respiratory distress syndrome.

## Data Availability

The data utilized for the preparation of the manuscript are included within the manuscript.

## References

[1] Bodley Scott R., Robb-Smith A. H. T. (1939). Histiocytic medullary reticulosis. *The Lancet*.

[2] Ramos-Casals M., Brito-Zerón P., López-Guillermo A., Khamashta M. A., Bosch X. (2014). Adult haemophagocytic syndrome. *The Lancet*.

[3] Griffin G., Shenoi S., Hughes G. C. (2020). Hemophagocytic lymphohistiocytosis: an update on pathogenesis, diagnosis, and therapy. *Best Practice & Research Clinical Rheumatology*.

[4] Yildiz H., Van Den Neste E., Defour J. P., Danse E., Yombi J. C. (2020). Adult haemophagocytic lymphohistiocytosis: a review. *QJM: An International Journal of Medicine*.

[5] Wong K.-F., Chan J. K. C. (1992). Reactive hemophagocytic syndrome-a clinicopathologic study of 40 patients in an oriental population. *The American Journal of Medicine*.

[6] Yang C.-W., Pan M.-J., Wu M.-S. (1997). Leptospirosis: an ignored cause of acute renal failure in Taiwan. *American Journal of Kidney Diseases*.

[7] Sripanidkulchai R., Lumbiganon P. (2005). Etiology of obscure fever in children at a university hospital in northeast Thailand. *The Southeast Asian Journal of Tropical Medicine and Public Health*.

[8] Rajagopala S., Singh N., Agarwal R., Gupta D., Das R. (2012). Severe hemophagocytic lymphohistiocytosis in adults-experience from an intensive care unit from North India. *Indian Journal of Critical Care Medicine : Peer-Reviewed, Official Publication of Indian Society of Critical Care Medicine*.

[9] Krishnamurthy S., Mahadevan S., Mandal J., Basu D. (2013). Leptospirosis in association with hemophagocytic syndrome: a rare presentation. *Indian Journal of Pediatrics*.

[10] Kodan P., Chakrapani M., Shetty M., Pavan R., Bhat P. (2015). Hemophagocytic lymphohistiocytosis secondary to infections: a tropical experience!. *Journal of Postgraduate Medicine*.

[11] Barman B., Lynrah K. G., Tiewsoh I., Jitani A., Ete T. (2016). Severe leptospirosis and secondary hemophagocytic syndrome: a rare case from Indian subcontinent. *Australasian Medical Journal (Online)*.

[12] Jevtic D., Djokic D., Redzic D., Aleksic D., Parezanovic M., Pasic S. (2018). Secondary hemophagocytic lymphohistiocytosis in a child with Leptospira infection: a case report. *The Turkish Journal of Pediatrics*.

[13] Guégan R., Camadro J. M., Saint Girons I., Picardeau M. (2003). Leptospira spp. possess a complete haem biosynthetic pathway and are able to use exogenous haem sources. *Molecular Microbiology*.

[14] Niller H. (2010). Myelodysplastic syndrome (MDS) as a late stage of subclinical hemophagocytic lymphohistiocytosis (HLH): a putative role forLeptospirainfection. A hypothesis. *Acta Microbiologica et Immunologica Hungarica*.

[15] Cagliero J., Villanueva S. Y. A. M., Matsui M. (2018). Leptospirosis pathophysiology: into the storm of cytokines. *Frontiers in Cellular and Infection Microbiology*.

[16] Grom A. A., Horne A., De Benedetti F. (2016). Macrophage activation syndrome in the era of biologic therapy. *Nature Reviews Rheumatology*.

[17] Fardet L., Galicier L., Lambotte O. (2014). Development and validation of the HScore, a score for the diagnosis of reactive hemophagocytic syndrome. *Arthritis & Rheumatology*.

[18] Otrock Z. K., Hock K. G., Riley S. B., De Witte T., Eby C. S., Scott M. G. (2017). Elevated serum ferritin is not specific for hemophagocytic lymphohistiocytosis. *Annals of Hematology*.

[19] Rodrigo C., Lakshitha De Silva N., Goonaratne R. (2014). High dose corticosteroids in severe leptospirosis: a systematic review. *Transactions of the Royal Society of Tropical Medicine and Hygiene*.

[20] Larroche C., Bruneel F., Andre M. (2000). Intravenously administered gamma-globulins in reactive hemaphagocytic syndrome. Multicenter study to assess their importance, by the immunoglobulins group of experts of CEDIT of the AP-HP. *Annales de medecine interne*.

[21] La Rosée P., Horne A., Hines M. (2019). Recommendations for the management of hemophagocytic lymphohistiocytosis in adults. *Blood*.

[22] Karpagam K. B., Ganesh B. (2020). Leptospirosis: a neglected tropical zoonotic infection of public health importance-an updated review. *European Journal of Clinical Microbiology & Infectious Diseases*.

[23] Warnasekara J., Koralegedara I., Agampodi S. (2019). Estimating the burden of leptospirosis in Sri Lanka; a systematic review. *BMC Infectious Diseases*.

